# Integrating microRNA target predictions for the discovery of gene regulatory networks: a semi-supervised ensemble learning approach

**DOI:** 10.1186/1471-2105-15-S1-S4

**Published:** 2014-01-10

**Authors:** Gianvito Pio, Donato Malerba, Domenica D'Elia, Michelangelo Ceci

**Affiliations:** 1 Department of Computer Science, University of Bari "Aldo Moro", Bari, I-70125, Italy; 2 Institute for Biomedical Technologies, CNR, Bari, I-70126, Italy

## Abstract

**Background:**

MicroRNAs (miRNAs) are small non-coding RNAs which play a key role in the post-transcriptional regulation of many genes. Elucidating miRNA-regulated gene networks is crucial for the understanding of mechanisms and functions of miRNAs in many biological processes, such as cell proliferation, development, differentiation and cell homeostasis, as well as in many types of human tumors. To this aim, we have recently presented the biclustering method HOCCLUS2, for the discovery of miRNA regulatory networks. Experiments on predicted interactions revealed that the statistical and biological consistency of the obtained networks is negatively affected by the poor reliability of the output of miRNA target prediction algorithms. Recently, some learning approaches have been proposed to *learn to combine *the outputs of distinct prediction algorithms and improve their accuracy. However, the application of classical supervised learning algorithms presents two challenges: *i) *the presence of only positive examples in datasets of experimentally verified interactions and *ii) *unbalanced number of labeled and unlabeled examples.

**Results:**

We present a learning algorithm that *learns to combine *the score returned by several prediction algorithms, by exploiting information conveyed by (only positively labeled/) validated and unlabeled examples of interactions. To face the two related challenges, we resort to a semi-supervised ensemble learning setting. Results obtained using miRTarBase as the set of labeled (positive) interactions and mirDIP as the set of unlabeled interactions show a significant improvement, over competitive approaches, in the quality of the predictions. This solution also improves the effectiveness of HOCCLUS2 in discovering biologically realistic miRNA:mRNA regulatory networks from large-scale prediction data. Using the miR-17-92 gene cluster family as a reference system and comparing results with previous experiments, we find a large increase in the number of significantly enriched biclusters in pathways, consistent with miR-17-92 functions.

**Conclusion:**

The proposed approach proves to be fundamental for the computational discovery of miRNA regulatory networks from large-scale predictions. This paves the way to the systematic application of HOCCLUS2 for a comprehensive reconstruction of all the possible multiple interactions established by miRNAs in regulating the expression of gene networks, which would be otherwise impossible to reconstruct by considering only experimentally validated interactions.

## Background

MicroRNAs (miRNAs) are small non-coding RNA molecules (~22 nucleotides in length) representing one of the most interesting class of gene regulators. Since their discovery in 1993 [1], the number of scientific reports on their functional characterization in a great variety of organisms has been growing at an impressive rate. They regulate cell cycle, modulate cell development and differentiation, are involved in the maintenance of cell homeostasis and apoptosis, and ultimately can influence the development and progression of many types of human tumors [2]. The growing amount of evidence of their key role in cancer and recent evidence of their presence in body fluids, such as serum and plasma, has further sparked the interest of the scientific community, emphasizing the possibility of using them as therapeutic targets and noninvasive biomarkers of diseases and of therapy response [3]. However, the full potential of their possible applications in the clinical domain depends on the understanding of their mechanisms and functions. Basically, miRNAs are post-transcriptional regulators that inhibit translation of messenger RNAs (mRNAs) by binding to complementary short sequences (6-8 nt in length), located inside the 3' untranslated regions (3'-UTRs) of transcripts. Depending on perfect or only partial complementarity between the miRNA seed sequence and its target site on the mRNA, the RNAi-induced silencing complex (RISC) associated to the miRNA can mediate the inhibition of translation initiation and/or mRNA decay [4]. More recent experimental evidence of miRNA functional targeting in gene promoter regions suggests that miRNAs may also play an important role in the transcriptional regulation of a great number of genes [5]. Moreover, the discovery of miRNAs' functional targeting in the 5' untranslated region (5'-UTR) and in the coding sequence (CDS) of mRNAs further complicates the understanding of their mechanisms.

According to current knowledge, the ability of miRNAs to act as a balance for a large variety of biological processes relies on their capacity to coordinately orchestrate cell signaling pathways by the multiple binding of many key effectors. Therefore, the identification of individual miRNA:mRNA interactions is not sufficient to catch the capacity of miRNAs to regulate complex gene networks. For this reason, much of the research in this field focuses on the development and application of biclustering algorithms [6,7].

In [7] we have recently proposed a method to identify significant miRNA:mRNA networks, by exploiting a novel biclustering algorithm. However, experiments performed on both experimentally validated and predicted interactions revealed that, although the latter provides a much larger amount of data to analyze, the significance of the networks obtained can be substantially affected by the reliability of the predictions. Indeed, prediction algorithms exhaustively analyze all the possible miRNA:mRNA pairs, searching for structural evidence that could suggest the existence of an interaction.

Examples of such algorithms are RNAhybrid [8], miRanda [9], TargesScan [10], DIANA-microT [11] and picTar [12]. Although these approaches are significantly cheaper than those based on experimental validation, results of these methods are in many cases uncorrelated to each other and their degree of overlap is poor. Their weakness depends on many factors, especially on the impossibility to incorporate in a single model all the possible interplaying variants that can influence the effects of the miRNA targeting, especially in mammals. Different results can also depend on the approach used and on the rules considered for the miRNA targeting, as well as on the type of resource of sequences they use as a reference dataset [13,14].

Furthermore, in [15] the authors showed that the reliability of such algorithms, in terms of precision and recall values computed against validated interactions, is, in general, very low. One of the approaches to overcome this issue consists in the combination of the predictions of several algorithms. In [16], some different approaches for combining predictions were compared, i.e. majority vote, ranking aggregation and Bayesian network classification. This last strategy represents one of the first attempts to exploit machine learning approaches to *learn to combine predictions *and, in this way, to identify a more reliable set of predicted interactions. In particular, the authors proposed the application of a supervised learning algorithm, i.e. a Bayesian network learner, to distinct sets of features considered in three well-known prediction algorithms, i.e. RNAhybrid, miRanda and TargesScan.

Although they are promising, existing machine learning solutions for learning to combine predictions are still at an embryonic stage [17]. For example, the applicability of the method proposed in [16] is limited to those scenarios in which a large number of both positive and negative examples is available. In general, when exploiting machine learning approaches to learn to combine interactions predicted, some issues have to be taken into account: *i) *Very few interactions are experimentally validated and can be considered as "stable" training examples. *ii) *Only positive examples of interactions are available, whereas negative examples are not generally available and, when available, their number is relatively small. *iii) *Prediction algorithms consider similar features and their simple combination can lead to the so-called collinearity problem [18].

All these issues are considered in this paper. In order to face *i)*, we propose a semi-supervised learning algorithm, which takes into account both (positively) labeled examples and the huge amount of unlabeled (unknown) instances during the learning phase. In order to overcome issue *ii)*, the proposed learning algorithm is able to learn from only positive examples. As for *iii)*, the collinearity problem can be alleviated by considering as features the scores (outputs) obtained by several prediction algorithms (instead of the original features), resorting to a solution which is similar to those adopted in meta-learning algorithms. The advantage of applying machine learning techniques to the outputs of several prediction algorithms consists in automatically adapting to unknown patterns of the outputs and associating more reliable prediction values when these patterns occur.

The proposed learning algorithm can be used either as a stand-alone software or in combination with the system HOCCLUS2 (an extension of the algorithm HOCCLUS [19]), in order to discover more complete and realistic miRNA:mRNA regulatory networks.

### Related work

The research reported in this paper has its roots in work which studies semi-supervised learning algorithms for learning from only positive examples. It also originates from work which studies the opportunity of combining the results of distinct miRNA target prediction algorithms, with the goal of obtaining more reliable predictions.

#### Learning a classifier from only positive and unlabeled training examples

The problem of learning a classifier in a semi-supervised setting (or in a transductive setting [20]) and, in particular, from only positively labeled examples, has already been investigated in many research papers. In general, as reported in [21], two main approaches have been followed in previous works. The most common consists in the identification of the most likely negative examples from the unlabeled set and in the application of a standard supervised learning algorithm [22-25]. This approach is sometimes extended to identify also additional positive examples from the unlabeled set [26].

The less common approach consists in assigning weights to unlabeled examples and then training a classifier which interprets them as weighted negative examples. This approach is used for instance in [27,28] and has been recently considered in [21], which inspired the method proposed in the present paper. The peculiarities of this last work are: first, it provides a principled way of choosing weights; second, it assigns a different weight to each unlabeled example, instead of assigning the same weight to every unlabeled example. However, contrary to our solution, the authors assume that each unlabeled example can be viewed as being both a weighted negative example and a weighted positive example, where the weights represent the probability that an unlabeled example is negative/positive. Since the two probabilities are not independent, this solution may generate redundancy in the representation. The second difference is that in [21], balancing is assumed between positive and unlabeled examples. This assumption does not hold in our case, where the number of miRNA:mRNA validated interactions is significantly lower than the number of possible miRNA:mRNA pairs. This last aspect motivates the use of the ensemble learning approach we have adopted, as explained in the rest of the paper.

#### Combining the output of miRNA target prediction algorithms

In [29], the authors identified two distinct approaches for data integration: the "low-level" approach, which directly deals with multi-factorial raw data and the "high-level" approach, which combines multiple same-type results from different studies. Following this classification, in [15] the authors evaluate a high-level solution that combines predictions provided by several existing algorithms. An interaction is considered reliable if at least *k *algorithms confirm it. In this case, however, the decision is taken on the basis of a simple counting of the algorithms that confirm a prediction. This means that this solution does not identify patterns of the outputs and does not adapt the final prediction to them. Finally, it is highly sensitive to the collinearity problem: algorithms that work on the same features will produce similar predictions, affecting the counting.

Similarly, StarBase [30], a recently developed database for exploring miRNA:mRNA interaction maps from argonauta CLIP-Seq (high-throughput sequencing of RNA isolated by crosslinking immunoprecipitation) and degradome-seq data (parallel analysis of RNA ends - PARE), intersects experimental results with predictions from six target prediction algorithms, to enhance precision and recall and identify miRNA-target regulatory relationships in six different organisms.

In [16], the authors evaluated the performance of single target prediction algorithms and of some high and low-level integration approaches to improve prediction accuracy. In particular, for high-level approaches they propose a simple majority voting solution and a ranking aggregation solution. As regards low-level approaches, the authors propose the application of a machine learning algorithm (i.e. Bayesian Network classification algorithm), which is able to provide a high level of adaptivity. The considered sets of features (low-level approach) are generated through combinatorially combining the sets of features taken into account by each single algorithm. Although the basic idea is similar to ours, the application of the machine learning algorithm to basic (possibly redundant) features could cause collinearity problems.

In addition to [16], in [31] the authors propose improving prediction capabilities through the application of machine learning solutions. However, in this case, a mixed high/low-level approach is followed. In particular, the authors propose the application of a Naïve Bayes classifier on a dataset of possible interactions represented by 57 structural features. The goal is to filter the output of the prediction algorithm miRanda, in order to decrease the amount of false positives. The problem of the absence of negative examples is solved by randomly generating dummy miRNAs and dummy interactions. The drawback of this solution is that the learned model is not deterministically determined and might be subject to some biases implicitly introduced in the artificially generated negative set.

Finally, in [32], the authors propose a high-level approach to learn a logistic regression model from the output of several miRNA target prediction algorithms. The proposed approach works in the classical supervised learning setting and does not exploit information from unlabeled examples (potential interactions) during the learning phase. This makes the approach difficult to apply when only few labeled interactions are available during learning and a huge amount of possible interactions have to be predicted. Moreover, the problem of negative examples does not apply in this case, since TarBase [33] + LCL [34], which contains both positive and negative examples, is used. Although the use of these datasets is, in this respect, beneficial, it limits the training set to a small number of interactions which is not comparable to the number of interactions we take into account during the learning phase (thousands vs. millions).

## Methods

The learning solution we present in this section is framed in the semi-supervised learning setting which, in addition to positive examples, takes advantage from unlabeled examples. Indeed, since we do not have negative examples in the training set, it becomes necessary to resort to this learning setting.

Before formally introducing the problem we intend to solve, some useful definitions are necessary. Let:

•  M and  G be the sets of miRNAs and mRNAs, respectively;

• x=⟨m,g⟩∈(M×G) be a (possible) interaction between miRNA *m *and mRNA *g*;

• *p_k_*(*x*) be the prediction score for the interaction *x *returned by the *k*-th target prediction algorithm, 1 *≤ k ≤ s*;

• *p*(*x*) = [*p*_1_(*x*), *p*_2_(*x*), ..., *p_s_*(*x*)] be the vector of prediction scores for the interaction *x*;

• *l*(*x*) be a function which returns 1 if *x *is a labeled (experimentally validated) interaction, 0 otherwise;

• *f*(*x*) be an ideal function which returns 1 if *x *represents a true interaction, 0 otherwise;

• L={x|x∈(M×G)∧l(x)=1} be the subset of labeled interactions;

• U=(M×G)-L be the subset of unlabeled interactions.

In our case, since only positive interactions are labeled, the following equation holds:

(1) P(f(x)=1|l(x)=1)=1

The goal is to learn a function $f^{\prime }\left(p\left(x\right)\right)$ which approximates the probability that *f*(*x*) = 1, that is $f^{\prime }\left(p\left(x\right)\right)\approx P\left(f\left(x\right)=\text{1}\right)$. As suggested in [21], it can be learned by exploiting (1) in the following steps:

$$\begin{array}{lll} P(l(x)=1) & = & P(f(x)=\text{1}\wedge l(x)=\text{1})\\ & = & P(l(x)=\text{1}|f(x)=\text{1}))\cdot P(f(x)=1) \end{array}$$

This means that

(2) $$f^{\prime }(p(x))\approx P(f(x)=1)=\frac{P(l(x)=1)}{P(l(x)=1|f(x)=1))}$$

In this equation, both the numerator and the denominator can be estimated by an ad-hoc probabilistic classifier specifically used for this purpose. In [21], this classifier is called *nontraditional classifier*. The following subsection is devoted to explaining how this classifier is used.

**Estimating P**(**l**(**x**) = **1**) **and P**(**l**(**x**) = **1***|***f**(**x**) = **1**))

In our work, the nontraditional classifier is learned through the LIBSVM algorithm [35] with Platt scaling [36], in order to get probability estimates. We choose a Support Vector Machine (SVM)-based algorithm mainly for the following reasons: *1) *they have a (relatively) good computational efficiency, especially in the prediction phase which is based on a very limited number of examples (support vectors); *2) *they are robust to noise and to feature redundancy [37]; *3) *their effectiveness (with Platt scaling) has already been evaluated and proved in the semi-supervised setting described in the paper which inspired our research [21]. However, it is noteworthy that every other algorithm that exhibits similar properties can be plugged into our framework.

LIBSVM is applied in order to solve the following problem:

*Given: *a set of training examples {(*p*(*x*), *l*(*x*))}*_x_*, where *p*(*x*) is the vector of prediction scores associated to the interaction *x *and *l*(*x*) (1 if the example is labeled, 0 otherwise) represents the class for the nontraditional classifier;

*Find: *a probability function $g:{\mathbb{R}}^{s}\to \mathbb{R}$ which takes as its input a vector of prediction scores *p*(*x*) and returns the probability that the interaction *x *is labeled. In this way, *g*(*p*(*x*)) *≈ P*(*l*(*x*) = 1).

In the way we use LIBSVM, we do not have testing examples and *g*(*p*(*x*)) represents the posterior class probability that a training example *p*(*x*) is classified as positive (that is, labeled), according to the optimal separating hyperplane of the nontraditional classifier.

As for the denominator, we assume that all labeled positive examples are taken completely randomly from all positive examples. Formally:

(3) P(l(x)=1|f(x)=1))=P(l(⋅)=1|f(⋅)=1))

In other words, P(l(x)=1| f(x)=1)) is independent of the specific interaction *x*. This assumption is essential for the purpose of learning from only positive examples and is coherent with the "selected completely at random" assumption in [21,38]. In this particular domain, this assumption could appear too much strong, since mRNA:miRNA pairs are generally not chosen randomly for biological validation. However, this happens in many other application domains, where examples are chosen on the basis of the trainer/expert's background knowledge. Moreover, it is noteworthy that this assumption is similar to that which is typically made for the classical classification task, where we assume that the underlying distribution of (labeled) positive and (labeled) negative examples in the training set is similar to that of the examples to be classified. This analogous assumption is typically considered also in the application of classifiers for prediction tasks in the biological domain (e.g. protein function prediction).

Assumption (3) allows us to use *g*(*p*(*x*)) also in the computation of P(l(x)=1|f(x)=1)). In particular, since a possible estimator of P(l(·)=1|f(·)=1)) is the average value of *g*(*p*(*x*)) for all labeled positive examples, we have:

(4) $$P(l(x)=\text{1}|f(x)=\text{1}))=P(l(\cdot )=1|f(\cdot )=1))\approx \frac{1}{\left|L\right|}\sum_{x\in L}g(p(x))$$

Differently from [21], in our case, we have to deal with the problem of unbalanced class distributions when learning the nontraditional classifier to obtain *g*(*p*(*x*)). Indeed, since the ratio between labeled and unlabeled examples is about 1/2000 (see Section "Results and Discussion"), LIBSVM would always learn a classifier which predicts all the interactions as unlabeled, independently of the considered interaction. In order to solve this problem, we resort to a sampling solution which is illustrated in the following.

### Ensemble learning g(·)

The sampling procedure considered in this work is similar to that used in bootstrap estimation of the value of an evaluation measure (e.g., predictive accuracy of a classifier) [39], as well as in some ensemble data mining methods, such as bagging [40], which combine multiple models to achieve better prediction accuracy than any of the individual models.

**Figure 1:**
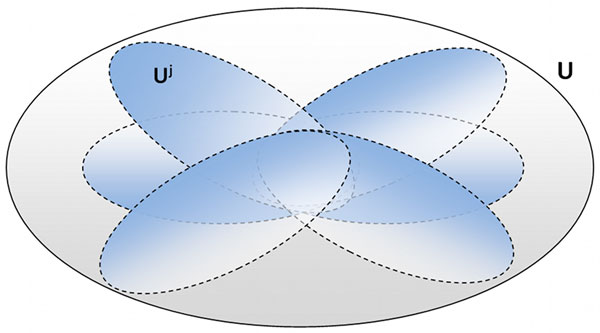
**Ensemble learning approach**. A graphical representation of the adopted ensemble learning approach. Each *U^j ^*contains a subset of the unlabeled examples.

More precisely, LIBSVM is run *K *times. At each execution, it is applied to the set of examples $L\cup U^{j}\left(j=\text{ 1},\text{2},\;.\;.\;.\;,K\right),$ that is, to all the labeled examples *L *and to a subset *U^j ^*of the unlabeled set *U *(Figure 1). In this way, we learn *K *nontraditional classifiers *g_j_*(*p*(*x*)), *j *= 1, ..., *K *that are combined to obtain *g*(*p*(*x*)).

The *K *subsets of unlabeled examples are built by randomly sampling, with replacement, *n *examples from *U*. The proportion of unlabeled examples in each *U^j ^*is $\frac{n}{\left|U\right|}$.

It is noteworthy that the *K *samples *U^j ^*are neither mutually exclusive nor exhaustive, i.e., they do not partition the original data set, so, for instance, even *K *= 10 samples with *n *= 0.1·|*U*| do not generally cover the entire set of unlabeled examples *U*. The probability that a particular unlabeled example is in ∪_*j *_*U*^*j *^is the following:

(5) $$\gamma =1-{\left(1-\frac{1}{\left|U\right|}\right)}^{n\cdot K}$$

When *K = |U|/n*, the above probability approximates 1 *- e*^-1 ^for large *|U|*, where *e *is Euler's number (≈ 2.7183). Since *e*^-1 ^≈ 0.368, this means that the expected number of unlabeled examples in $\cup_{j}U^{j}$ is 63.2% of those in *U*.

Since we are interested in covering a given proportion *γ *of negative examples (e.g. 90%), we rewrite (5) in terms of the expected number of samples necessary to cover at least *γ *unlabeled examples:

(6) $$K=\frac{1}{n}\cdot \frac{\log\left(1-\gamma \right)}{\log\left(1-\frac{1}{\left|U\right|}\right)}$$

Differently from data partitioning, which is affected by only one parameter *K *(the number of partitions), the data sampling procedure used in this work is controlled by two parameters: *n *and *γ*. The first parameter represents the number of unlabeled examples in each sample and can be reasonably chosen on the basis of the number of labeled examples, so that the unbalancing problem is mitigated. The second parameter represents the percentage of unlabeled examples we intend to cover.

Once the *K *classifiers are learned, each classifier *g_j_*(*p*(*x*)) is applied to obtain an estimate of *P*(*l*(*x*) = 1) for all the examples in $U^{j}$. Since the same unlabeled example can belong to more than one sample, the following equation is used:

(7) $$g\left(p\left(x\right)\right)=\underset{\left\{j|x\in U^{j}\right\}}{\mathrm{average}}g_{j}\left(p\left(x\right)\right)$$

### Ensemble learning $f^{\prime }\left(\cdot \right)$

In order to identify the function $f^{\prime }\left(p\left(x\right)\right)$, a straightforward solution would be to directly apply Equation (2). However, as empirically proved in [21], a more effective solution consists in the computation of a weight for each example and in training a further (traditional) classifier.

Specifically, we compute the probability that an unlabeled example *x *represents a positive example as:

(8) $$\begin{array}{ccc} P(f(x)=1|l(x)=0) & = & \frac{P(l(x)=0,f(x)=1)P(f(x)=1)}{P(l(x)=0)}\\ & = & \frac{\left[1-P(l(x)=1|f(x)=1)]P(f(x)=1\right)}{1-P(l(x)=1)}\\ & = & \frac{[1-P(l(x)=1|f(x)=1)]\cdot \frac{P(l(x)=1)}{P(l(x)=1|f(x)=1)}}{1-P(l(x)=1)} \end{array}$$

which, according to Equation (4), can be approximated to:

(9) $$P\left(f\left(x\right)=1|l\left(x\right)=0\right)\approx \frac{1-c}{c}\cdot \frac{P\left(l\left(x\right)=1\right)}{1-P\left(l\left(x\right)=1\right)}$$

where $c=\frac{1}{\left|L\right|}\sum_{x\in L}g\left(p\left(x\right)\right)$ and *P*(*l*(*x*) = 1) is approximated to *g*(*p*(*x*)).

The training set for the traditional classifier which is in charge of learning *f'*(*p*(*x*)) is then built as follows:

(10) $$training\text{\_}label\left(x\right)=\left\{\begin{array}{c} +\;if\;x\in L\\ +\;if\;x\in U\wedge P\left(f\left(x\right)=1|l\left(x\right)=0\right)\geq P\left(f\left(x\right)=0|l\left(x\right)=0\right)\\ -\;otherwise \end{array}\right)$$

(11) $$weight\left(x\right)=\left\{\begin{array}{c} 1.0\;\;\;\;\;\;\;\;\;\;\;if\;x\in L\\ P\left(f\left(x\right)=1|l\left(x\right)=0\right)\;\;\;if\;x\in U\wedge P\left(f\left(x\right)=1|l\left(x\right)=0\right)\geq P\left(f\left(x\right)=0|l\left(x\right)=0\right)\\ 1-P\left(f\left(x\right)=1|l\left(x\right)=0\right)\;otherwise \end{array}\right)$$

$f^{\prime }\left(p\left(x\right)\right)$ is learned by applying a variant of LIBSVM (http://www.csie.ntu.edu.tw/~cjlin/libsvmtools/#weights_for_data_instances) which allows us to specify a weight for each example. In general, in this algorithm, the weight assigned to an example represents the cost of misclassifying it, which is then exploited in the SVM optimization process. In our case, the strategy adopted to compute the weight exploits the probability (Equation (11)) that the assigned label (Equation (10)) is correct. In this way, intuitively, the misclassification cost for a given example will be proportional to the confidence we have in the assigned label.

The strategy we adopt for learning the traditional classifier differs from that adopted in [21], which, as previously stated, represents each unlabeled example as both a positive example with weight $P\left(f\left(x\right)=\text{1}|l\left(x\right)=0\right)$ and a negative example with weight $\text{1}-P\left(f\left(x\right)=\text{1}|l\left(x\right)=0\right).$ This generates redundancy in the representation and possibly prevents the algorithm from learning a good separating hyperplane.

Similarly to the nontraditional classifier, also in this case, we solve the class unbalancing problem (in this case, unbalancing is between positive examples and negative examples, instead of labeled and unlabeled examples), by resorting to the same bagging procedure described in the previous section. The procedure in this case is still necessary since the number of true miRNA:mRNA interactions (positive examples) is significantly smaller than the number of remaining possible miRNA:mRNA pairs (negative examples).

A final remark is made to explain how our algorithm faces the collinearity problem [18]. Collinearity is the problem according to which if some features are (nearly) linearly dependent on the others, a predictive model may not be well estimated. In our case, it is possible that used prediction algorithms consider highly overlapping characteristics and, although we work on their outputs and not directly on the characteristics, the collinearity problem may still be present. In this respect, an important advantage introduced by our algorithm is that it automatically adapts to highly redundant characteristics and does not require a preliminary feature selection step. This important property is achieved through the use of an SVM-based solution, which, as in other scientific fields, has proved to be robust to noise and to highly redundant features [37].

## Results and discussion

In this section, we present the considered datasets, define the experimental setting, introduce evaluation measures and present a discussion about obtained results.

### Datasets

In order to evaluate our approach, we have considered as data sources a set of experimentally verified miRNA:mRNA interactions, i.e. miRTarBase [41], as well as the set of miRNA target predictions in mirDIP [15]. Interactions from miRTarBase have been used as positive/labeled examples and interactions from mirDIP, but not present in miRTarBase, have been considered as unlabeled examples. In the learning phase, examples are represented according to the standardized scores returned by the algorithms that mirDIP integrates (standardization is performed by mirDIP; although standardization makes scores comparable for the human expert, our algorithm does not strictly require it). Furthermore, we have used TarBase [33] as a testing set because it contains both positive and negative experimentally verified miRNA:mRNA interactions. It is noteworthy that TarBase can also be used in the training phase. However, in this work we have decided to use it in the testing phase, in order to provide good estimates of the algorithm performance on a valid independent test set.

The miRTarBase ver. 3.5 dataset (http://mirtarbase.mbc.nctu.edu.tw/) contains 4,867 experimentally verified miRNA-target interactions between 729 miRNAs and 2,789 target genes among 17 species. miRNA-target interactions are collected by manually surveying pertinent literature after applying text mining techniques to filter research articles related to functional studies of miRNAs. Generally, the collected interactions are validated experimentally by reporter assay, western blot, or microarray experiments with overexpression or knockdown of miRNAs. In our study, we only consider the human dataset.

The mirDIP dataset (http://ophid.utoronto.ca/mirDIP/) is an integrated database which includes miRNA target predictions of twelve different datasets. In this study, we consider only the predictions which refer to the 3' UTR region, i.e. those returned by DIANA-microT [11], microCosm [42], miRanda [9], picTar 4-way and picTar 5-way [12], PITA All Targets and PITA Top Targets [43], TargetScan Conserved and TargetScan Non-Conserved [10] and RNA22 3' UTR [44]. As anticipated in the previous section, the high redundancy among the features considered by these datasets motivates the SVM-based solution. The mirDIP dataset used in our experiments contains approximately 5 million predicted interactions between 934 miRNAs and 30,875 mRNAs. The number of predictions returned by each algorithm is reported in Table 1.

**Table 1:** Number of predictions returned by each considered algorithm in mirDIP.

Algorithm	N. Predictions
DIANA-microT	1,434,409
microCosm	568,103
miRanda	956,667
picTar 4-way	56,232
picTar 5-way	17,226
PITA All Targets	4,010,550
PITA Top Targets	208,940
TargetScan Conserved	189,078
TargetScan Non-Conserved	1,457,487
RNA22	264,633

TarBase 6.0 (http://www.microrna.gr/tarbase) is the largest available manually curated target database, indexing more than 65,000 miRNA-gene interactions. The database includes targets derived from gene-specific and high throughput experiments.

### Experimental setting

**Figure 2:**
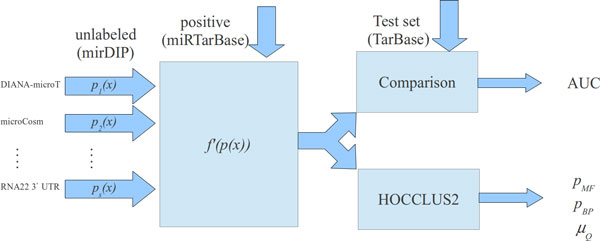
**Two experimental settings**. After the model $f^{\prime }\left(\right)$ is learned, it is used for comparison with other strategies and for the identification of regulatory networks through the use of HOCCLUS2.

The main goal of the experiments is twofold: *a) *To evaluate the accuracy of the predictions provided by our algorithm by taking as input unlabeled (a large set of predicted miRNA:mRNA interactions) and positive examples. *b) *To evaluate whether our algorithm can improve the identification of meaningful regulatory networks. Indeed, as shown in [7], working with a large set of interactions does not always lead to the improvement of the quality of the obtained results. On the contrary, especially when the input data are affected by a huge amount of false positives and false negatives, the significance of the obtained regulatory networks may be compromised. The complete workflow of the experiments is reported in Figure 2.

As for *a)*, we have used TarBase [33] as a test set, which, although limited in the number of included interactions, contains both positive and negative examples. In order to guarantee a fair comparison, we have removed from TarBase all the examples that are also reported in miRTarBase, since they could give an advantage to our approach because used in the learning phase. We also removed inconsistent examples, that is, interactions labeled as both positive and negative. At the end, the considered test set contains 29,091 positive examples and 3,910 negative examples of interactions.

In this study, we compare our approach with several alternative solutions:

• *Single prediction algorithms *(DIANA-microT, microCosm, miRanda, picTar 4-way, picTar 5-way, PITA All Targets, PITA Top Targets, TargetScan Conserved, TargetScan Non-Conserved, RNA22 3' UTR).

• *Score averaging *(**SA**): a simple algorithm that equally weights the contribution of each single prediction algorithm.

• *Score averaging - three best *(**SA-3B**): an algorithm that equally weights the contribution of the best three prediction algorithms (TargetScan Conserved, PITA Top Hits and picTar 5-way), according to [15].

• *Weighted score averaging - three best *(**WSA-3B**): an algorithm that weights the contribution of the best three prediction algorithms (TargetScan Conserved, PITA Top Hits and picTar 5-way). Weights are proportional to the reliability (computed on the basis of the F-Score) of each algorithm, according to [15].

The last two solutions have been proposed in [7]. It is noteworthy that these combination strategies can be considered a finer variant of the majority vote [16] and counting [15] strategies, since the scores of the predictions are taken into account.

As regards *b)*, in order to identify regulatory networks, we used the system HOCCLUS2 [7], which is based on a biclustering algorithm that has been proved to be a valid tool for supporting biologists in this task. In particular, HOCCLUS2 is able to extract cohesiveness-preserving biclusters, when compared with competitive approaches, containing mRNAs which are statistically more functionally similar than mRNAs which belong to different biclusters. HOCCLUS2 requires two parameters: *α*, which is the minimum cohesiveness value (see next section for details about the cohesiveness) a bicluster must satisfy after merging, and *β*, which is the minimum score that must be associated to an interaction to be considered as reliable. Experiments have been conducted with different values of *α *and *β*. This is necessary in order to understand if, with the proposed approach, the quality of discovered interaction networks depends on their values.

Coherently with the experimental setting *a)*, HOCCLUS2 has been applied to different data sets of predicted interactions, obtained by applying our approach and three other combination strategies, that is, SA, SA-3B, WSA-3B.

### Evaluation measures

In order to evaluate the accuracy of the predictive models learned by the proposed algorithm we consider the Area Under the ROC Curve (AUC) [45].

In order to evaluate the quality of extracted biclusters, we use the average biclustering cohesiveness and a statistical measures based on the Student's *t*-test. The average biclustering cohesiveness measures the average strength of the intra-bicluster connections: $\mu_{q}\left(L_{j},A\right)=\frac{1}{\sum_{C_{i}\in L_{j}}|C_{i}|}\sum_{C_{i}\in L_{j}}|C_{i}|q\left(C_{i},A\right)$, where *L_j _*is the set of biclusters obtained at the *j*-th hierarchy level, *C_i _*is a bicluster, *|C_i_| *is the number of miRNAs and mRNAs in *C_i _*and *q *is defined as $q\left(C,A\right)=\frac{\sum_{x\in C}\left(miRNA\right)\sum_{y\in C}\left(mRNA\right)\;A_{x,y}}{\left|C^{\left(miRNA\right)}|\cdot |C^{\left(mRNA\right)}\right|}$. In the definition of *q*(*C, A*), *C*^(*miRNA*) ^is the set of miRNAs in the biscluster *C, C*^(*mRNA*) ^is the set of mRNAs in *C *and *A_x,y _*is the score of the interaction (in mirDIP) between the miRNA *x *and the mRNA *y*. This function measures the weighted (i.e. by considering the score of the interactions) percentage of interactions in a bicluster, normalized by the maximum number of possible interactions.

In addition to *μ_q_*(), we also use an evaluation measure which is based on the statistical properties of the obtained biclusters. In particular, we use the independent two-sample Student's *t*-test to evaluate the null hypothesis $H_{0}:\mu_{0}^{\prime }\left(L_{j}\right)=\mu^{\prime }\left(L_{j}\right)$ against the alternative hypothesis $H_{1}:\mu_{0}^{\prime }\left(L_{j}\right)=\mu^{\prime }\left(L_{j}\right)$, where $\mu_{0}^{\prime }\left(L_{j}\right)$ is the average intra-bicluster functional similarity $\mu_{0}^{\prime }\left(L_{j}\right)=\frac{1}{\left|L_{j}\right|}\sum_{C\in L_{j}}\mu_{0}\left(C\right),\mu^{\prime }\left(L_{j}\right)$ is the average inter-bicluster functional similarity defined as

(12) $$\mu^{\prime }(L_{j})=\frac{1}{\left|L_{j}|\cdot (|L_{j}|-1\right)}\sum_{C_{1}\in L_{j},C_{2}\in L_{j},C_{1}\neq C_{2}}\left(\frac{\sum_{x_{1}\in (C_{1}^{\left(mRNA\right)}\backslash C_{2}^{\left(mRNA\right)}),x_{2}\in (C_{2}^{\left(mRNA\right)}\backslash C_{1}^{\left(mRNA\right)})}SimGIC(x_{1},x_{2})}{\left|C_{1}^{\left(mRNA\right)}\backslash C_{2}^{\left(mRNA\right)})|\cdot |C_{2}^{\left(mRNA\right)}\backslash C_{1}^{\left(mRNA\right)}\right|}\right)$$

and

(13) $$\mu_{0}\left(C\right)=\frac{1}{|C_{r}|\cdot \left(|C_{r}|-1\right)}\underset{x_{1}\in C_{r},\in x_{2}\in C_{r},x_{1}\neq x_{2}}{\sum }SimGIC\left(x_{1},x_{2}\right)$$

In (12) and (13) *SimGIC *[46] is a semantic similarity measure computed between two genes, according to the UniProt Homo sapiens GO annotations.

The lower the *p*-value (obtained by the two-sample Student's *t*-test), the higher the difference between the average intra-functional similarity and the average inter-functional similarity. We use both GO Biological Process (BP) and GO Molecular Function (MF) hierarchies to compute *SimGIC*. Henceforth we will refer to the *p*-values computed on BP and MF as *p_BP _*and *p_M F_*, respectively.

### Results

In Table 2 we report AUC results obtained by our approach with different values of the sampling parameters *n *and *γ*. As expected, the higher the value of *γ *the better the performance of the algorithm, since a larger amount of unlabeled examples is considered. On the other hand, setting *γ *to a value which is close to 1 leads to an infinite number of samples (according to Equation (6), $\underset{\gamma \to 1^{-}}{\lim}\frac{1}{n}\cdot \frac{\log\left(1-\gamma \right)}{\log\left(1-\frac{1}{\left|U\right|}\right)}=+\infty$). This means that *γ *has to be set by keeping in mind a good balance between effectiveness and efficiency.

**Table 2:** AUC results obtained by our approach with different values of the parameters *n *and *$\gamma$*.

n	*γ*				
	
	0.5	0.6	0.7	0.8	0.9
**5,000**	0.582	0.599	0.614	0.629	0.647
**10,000**	0.585	0.601	0.616	0.634	0.649
**15,000**	0.579	0.594	0.601	0.625	0.640

Moreover, changing the number of unlabeled examples in each sample *n *does not lead to a significant difference in the results, although results with *n *= 10,000 outperform those obtained with other values of *n*. For the experiments reported in the rest of the paper we selected the parameters which let us obtain the best results, i.e. *n *= 10,000 and *γ *= 0.9.

In Table 3 we report AUC results for all the considered algorithms/approaches. They clearly show that results obtained with our approach outperform those obtained with all the single prediction algorithms. This confirms previous findings [15] and, in particular, that combined approaches, in general, are able to outperform single algorithms. The only algorithm which is able to produce results which are comparable to those obtained by our approach is PITA All Targets. This result is motivated by the high number of interactions in this dataset (see Table 1), which make the predictor more informed about TarBase interactions (test set). However (as we will argue later), it is not able to generate high-quality biclusters, due to the large amount of false positives it predicts.

**Table 3:** AUC TarBase results.

Algorithm/Strategy	AUC
DIANA-microT	0.500
microCosm	0.519
miRanda	0.544
picTar 4-way	0.509
picTar 5-way	0.507
PITA All Targets	**0.640**
PITA Top Targets	0.528
TargetScan Conserved	0.536
TargetScan Non-Conserved	0.563
RNA22	0.509

SA-3B	0.543
SAWA-3B	0.543
SA	**0.608**
Our Approach	**0.649**

The upper part of the table contains AUC results of single prediction algorithms. The lower part reports AUC results obtained by combination strategies.

**Figure 3:**
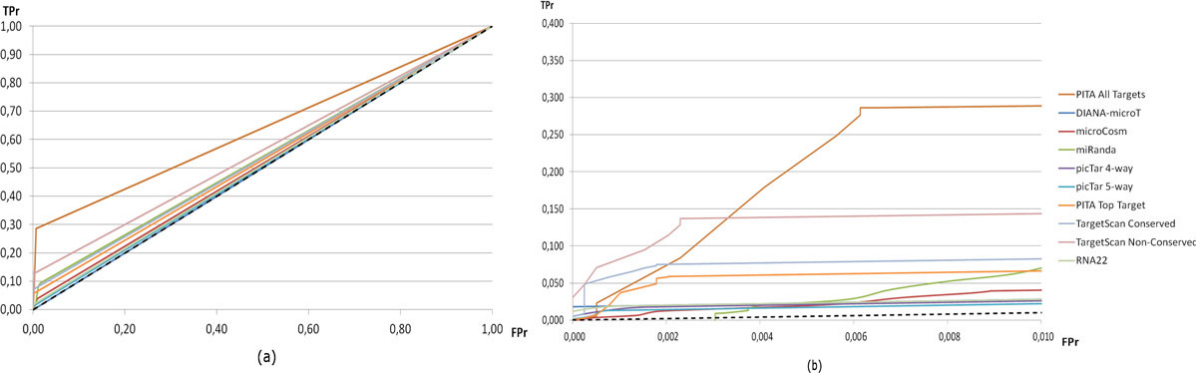
**Performance of single prediction algorithms**. (a) ROC curves for single prediction algorithms. (b) A zoom on the bottom left corner.

Figure 3 provides additional details on results reported in Table 3. In fact, as it can be seen, the most conservative algorithms are those for which the ROC curve provides good True Positive rate (TPr) values for small False Positive rate (FPr) values (concentrating on the bottom-left corner of the chart, see Figure 3(b)). This provides a way to refine what is suggested in [15]. In particular, one might suggest using the prediction algorithms as follows: *1) *when looking for confirmatory evidence of a particular interaction, it is better to use a database with superior recall, such as TargetScan Conserved or TargetScan Non-Conserved (which have high TPr for low FPr). Contrary to results reported in [15], in our case, microCosm does not appear to satisfy these properties (see Figure 3(b)). *2) *When identifying any possible targets for a particular microRNA to form the basis for in vitro or in vivo experiments, it would be best to consult a conservative algorithm, that is, an algorithm which returns a limited number of (possibly reliable) interactions, such as picTar 5-way (see Table 1). *3) *When finding in silico evidence for an interaction of a microRNA and a gene of a certain family or function, it is best to use an algorithm with a more even balance between precision and recall such as PITA All Targets and TargetScan Non-Conserved (which have high AUC, graphically, the area under the curve). This last conclusion is different from that drawn in [15], where the use of PITA Top Targets is suggested. A possible motivation for differences between our conclusions and those reported in [15] can be the use of a different test set (we use TarBase, while in [15] results of 15 publicly available microRNA over-expression/knockdown experiments are considered) and different evaluation measures (we use AUC instead of fixed threshold-based precision and recall).

**Figure 4:**
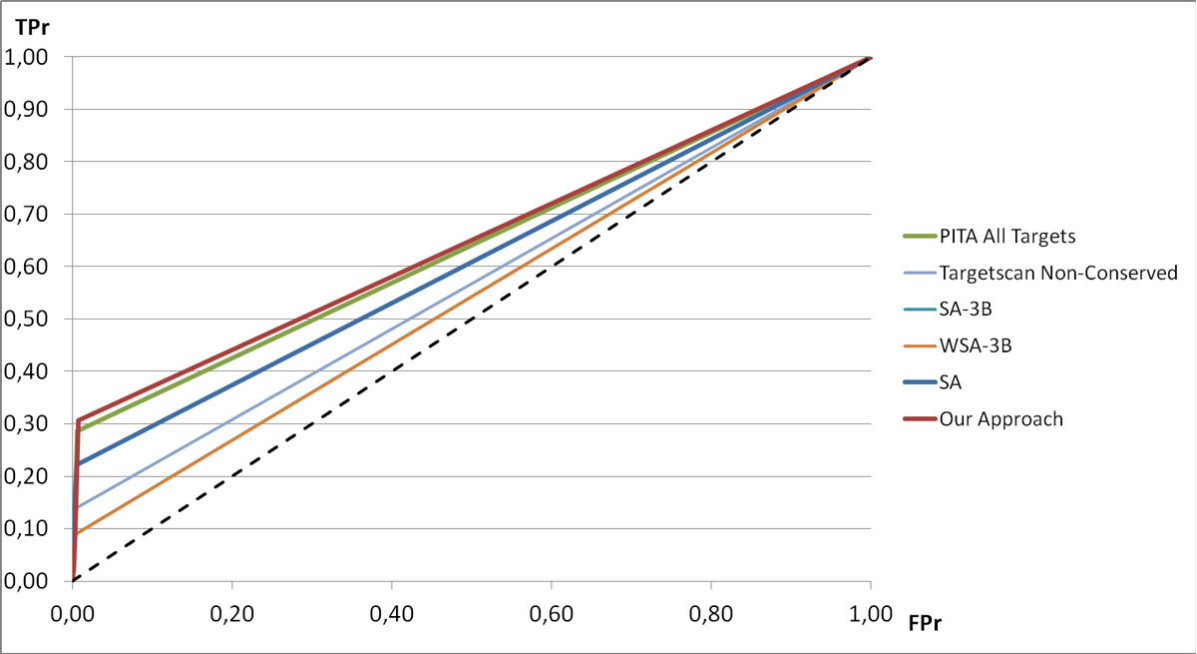
**ROC curves for the algorithms with the best AUC value**.

If we consider combined approaches, we see that, in general, they are able to reach predictive capabilities (in terms of AUC) which are comparable to the best prediction algorithms (see Figure 4). They are also able to work well for low FPr values. If we consider the specific case of our approach, it is able to outperform of a great margin all the combined approaches and all the prediction algorithms.

The results reported in Table 4 refer to the problem of identifying regulatory networks in the form of biclusters extracted by HOCCLUS2. In particular, Table 4 reports the quantitative results obtained for the first hierarchy level, the last hierarchy level and the best hierarchy level (according to *p_BP _*and *p_MF _*values), using different values of *α *and *β*. Observing the best level, it is possible to see that the proposed approach always leads to the identification of at least one level with very low *p_BP _*and *p_MF _*values, independently of the choice of the parameters of HOCCLUS2. Moreover, comparing the results with those obtained with the SA approach (which is the best among the considered competitors), it is noteworthy that our approach always lets HOCCLUS2 extract a smaller number of biclusters, grouping less miRNAs and mRNAs. This is due to the fact that our approach is able to better filter out false positives and allows HOCCLUS2 to focus only on more reliable interactions (lower FPr, for a given TPr).

**Table 4:** Quality of biclusters obtained by HOCCLUS2.

*α*	*β*	$\underset{\left(\text{mRNA}/\text{miRNA}\right)}{N}$	level 1				max level					best level				
			
			#cc	*p_MF_*	*p_BP_*	*μ_q_*	lev	#cc	*p_MF_*	*p_BP_*	*μ_q_*	lev	#cc	*p_MF_*	*p_BP_*	*μ_q_*
**Predictions - SA-3B**																

0.1							9	56	0.000	0.000	0.12	2	350	0.000	0.000	0.41
							
0.2	0.3	5698/612	700	1.000	1.000	0.49	7	183	0.000	0.000	0.24	3	210	0.000	0.000	0.31
							
0.3							5	355	1.000	1.000	0.36	1	700	1.000	1.000	0.49

0.1							8	41	0.411	0.331	0.11	3	155	0.004	0.009	0.32
							
0.2	0.4	4735/607	619	1.000	1.000	0.52	7	144	0.006	0.001	0.24	7	144	0.006	0.001	0.24
							
0.3							6	274	1.000	1.000	0.35	1	619	1.000	1.000	0.52

0.1							8	34	0.284	0.273	0.12	4	77	0.345	0.167	0.27
							
0.2	0.5	3337/572	599	1.000	1.000	0.58	7	101	0.315	0.146	0.23	5	108	0.257	0.112	0.26
							
0.3							6	202	1.000	0.221	0.34	5	205	1.000	0.206	0.35

**Predictions - WSA-3B**																

0.1							9	57	0.023	0.005	0.11	2	379	0.000	0.000	0.41
							
0.2	0.3	6209/618	758	1.000	1.000	0.50	7	194	0.016	0.004	0.25	3	221	0.001	0.000	0.31
							
0.3							6	374	1.000	1.000	0.36	1	758	1.000	1.000	0.50

0.1							7	42	0.434	0.206	0.11	4	58	0.094	0.016	0.21
							
0.2	0.4	5122/601	667	1.000	1.000	0.54	6	145	0.096	0.004	0.24	5	148	0.053	0.004	0.25
							
0.3							5	273	1.000	1.000	0.34	1	667	1.000	1.000	0.54

0.1							8	35	0.311	0.346	0.12	3	156	0.151	0.263	0.37
							
0.2	0.5	3653/570	622	1.000	1.000	0.60	7	105	0.221	1.000	0.24	3	168	0.123	0.298	0.38
							
0.3							6	205	0.374	1.000	0.36	2	314	0.256	1.000	0.50

**Predictions - SA**																

0.2	0.3	8723/599	294	0.140	0.080	0.43	7	58	0.262	0.253	0.22	1	294	0.140	0.080	0.43
							
0.3							5	182	0.328	0.176	0.38	1	294	0.140	0.080	0.43

0.2	0.4	7772/620	1608	1.000	1.000	0.50	9	216	0.001	0.006	0.22	3	416	0.008	0.000	0.33
							
0.3							7	604	0.000	0.000	0.34	2	830	0.000	0.000	0.42

0.2	0.5	4336/627	1038	1.000	1.000	0.58	9	96	0.399	0.364	0.22	4	148	0.286	0.261	0.31
							
0.3							7	283	1.000	1.000	0.35	2	522	1.000	0.228	0.47

**Predictions - Our Approach**																

0.1							8	40	0.013	0.020	0.11	2	444	0.000	0.000	0.52
							
0.2	0.3	2379/614	888	1.000	1.000	0.69	7	143	0.000	0.000	0.24	2	444	0.000	0.000	0.52
							
0.3							6	268	0.002	0.001	0.37	3	309	0.000	0.000	0.42

0.1							8	25	0.175	0.080	0.12	3	148	0.000	0.000	0.39
							
0.2	0.4	1626/544	591	0.404	1.000	0.77	7	84	0.015	0.001	0.24	3	152	0.000	0.000	0.39
							
0.3							6	161	0.001	0.377	0.38	2	298	0.000	0.001	0.57

0.1							8	16	0.079	0.053	0.12	3	105	0.000	0.000	0.43
							
0.2	0.5	1245/467	417	0.361	0.244	0.83	7	53	0.000	0.000	0.24	3	105	0.000	0.000	0.43
							
0.3							7	104	0.000	0.000	0.39	4	110	0.000	0.000	0.42

N represents the number of biclustered mRNAs and miRNAs. #cc is the number of biclusters. *lev *represents the level number. The "best" level is the level with the lowest $\frac{p_{MF}+p_{BP}}{2}$ value.

Different considerations can be drawn from the analysis of results obtained by HOCCLUS2 on the single prediction algorithm which shows the best AUC value, that is, PITA All Targets. In fact, results with *α *= 0.2 and *β *= 0.5 (best configuration of HOCCLUS2) lead to *p_MF_, p_BP _*and *μ_q _*results which are, for the levels of the hierarchy whose results can profitably used by the expert (*≥ *2), not comparable to those obtained by combined approaches (see Table 5). Indeed, the higher the level, the worse the performance in terms of all the considered evaluation measures. This is motivated by the high number of false positives of PITA All Targets which leads to a degeneration of the quality of the extracted interaction networks. It is noteworthy that this issue appears only during this evaluation, since HOCCLUS2 works on the whole set of interactions of the dataset, whereas the AUC value is computed only on the interactions that are reported in TarBase. For these reasons, in the following we will focus only on the results obtained by combined approaches.

**Table 5:** Quality of biclusters obtained by HOCCLUS2 on PITA All Targets

level	*p_MF_*	*p_BP_*	*μ_q_*
1	0.149	0.155	0.563
2	0.284	0.268	0.469
3	0.384	0.337	0.384
4	0.421	0.373	0.298

In Table 6, we report the distribution of biclusters with *p_BP _≤ *0.05 over different levels of the hierarchy. From the table it is possible to see that, for all levels of the hierarchy, the only approach that is comparable to ours is SA which, however, does not reach the same number of biclusters with *p_BP _≤ *0.05. In particular, at lower and higher levels of the hierarchy, the difference between the results obtained in SA and our approach increases. Moreover, it is noteworthy that there is no degeneration (possibly due to the presence of false positives and false negatives) introduced in the merging phase of HOCCLUS2. This is motivated by the more reliable predictions provided by our algorithm with respect to other approaches.

**Table 6:** Distribution of biclusters with *p_BP _≤ *0.05 over different levels of the hierarchy.

	% of biclusters with p_BP _≤ 0.05			
**Hierarchy** **Level**	**SA-3B**	**WSA-3B**	**SA**	**Our Approach**

L1	0.0	0.0	26.5	32.6
L2	34.0	33.0	34.5	36.4
L3	34.0	33.9	35.1	40.0
L4	37.6	33.3	37.2	37.1
L5	38.9	32.1	40.2	41.4
L6	38.2	30.8	40.4	42.6
L7	38.6	30.5	40.8	43.4
L8	-	-	40.2	-
L9	-	-	40.6	-

Results are obtained with *α *= 0.2, *β *= 0.5.

### Evaluation of biological consistency of extracted biclusters

In this subsection we report some examples of extracted biclusters and discuss the results of their biological analysis. Biclusters have been selected according to the statistical ranking returned by HOCCLUS2. Biological consistency of biclusters has been evaluated by considering: *i) *structural and functional properties of miRNAs; *ii) *functional clustering and pathway mapping of target genes; *iii) *information available from the literature supporting the functional miRNA:mRNA relationships suggested by the biclustering results. A series of web resources, such as miRBase [42] and GeneCards [47], have been used to retrieve information on gene families, gene clusters and gene functions. Set Distiller [48], from the GeneCards tool suite, and Reactome [49] have been used for functional clustering and pathway mapping of miRNA target genes, respectively. Other resources, such as STRING [50], have been used for network-based enrichment analysis of target genes, on the basis of known and predicted protein interactions and functional relationships.

#### Quantitative comparison of HOCCLUS2 results on the miR-17-92 gene cluster family: new approach vs SA-3B

In our previous work [7], HOCCLUS2 was tested by using two benchmarks, experimentally validated miRNA:mRNA interactions, i.e., miRTarBase [51], and miRNA target site predictions from mirDIP [15].

In order to prove the effectiveness of the new approach on mirDIP data, we focus on biclusters containing members of the miR-17-92 gene cluster family and its paralogs, miR-106b-25 and miR-106a-363. They have been chosen because of the wealth of information available from the current literature, which can be exploited to verify whether the obtained biclusters suggest biologically realistic miRNA:mRNA regulatory networks. Furthermore, different types of experimental evidence suggest that miRNAs belonging to miR-17-92 may perform specific functions, either individually or in combination, in a coordinated rather than in an additive manner [52]. Due to this peculiar feature, the miR-17-92 gene cluster family is, among all the possible candidates, the best for proving the ability of HOCCLUS2 to discover miRNA context-specific regulatory modules at different granularity levels, according to the hierarchy of biclusters.

Looking at Tables 3 and 4, it would seem to be natural to compare our results with those of SA, since it shows a good AUC value as well as good *p_BP _*and *p_MF _*values. However, a preliminary qualitative analysis of the extracted biclusters revealed that significant biclusters (in terms of *p_BP _*and *p_MF_*) appear mainly at high levels in the hierarchy. Analyzing such biclusters with Reactome and STRING, we notice that they do not show the expected biological consistency. Furthermore, they group too many miRNAs (also belonging to different families) in the same bicluster. Although in principle this is a coherent behavior, such situation does not allow the researchers to distinguish between specific and general interactions at different granularity levels, which is the main goal of the task of discovering interaction networks organized in a hierarchy. This behavior is mainly due to the fact that SA averages the scores of all the algorithms, including also unreliable predictions. The consequence is that this algorithm tends to "flatten" the score of all the interactions and, consequently, to affect the possibility that HOCCLUS2 focuses only on reliable interactions. For these reasons, we compare our results with those obtained with the SA-3B setting that, although generally showing worse results in terms of AUC, *p_BP _*and *p_MF_*, allowed us (also in the experiments conducted in our previous work [7]) to perform an analysis starting from the lowest (most specific) levels of the hierarchy.

Hereafter, the whole set of biclusters obtained on the basis of SA-3B setting and with the new approach will be referred to as *mirDIP-A *and *mirDIP-B*, respectively. Comparison takes into account the number of biclusters, their biological statistical significance (*p_BP _*value) and cohesiveness values (*μ_q_*). In particular, we focus on biclusters extracted by HOCCLUS2 with *α *= 0.2 and *β *= 0.5.

As shown in Table 7, the results obtained in the two experiments are significantly different. In particular, they show a considerable improvement of the system performance with the new approach with respect to the SA-3B setting. Indeed, among *mirDIP-A *biclusters, the total number of biclusters containing miR-17 is 13 and, among them, only one (about 8% of the total) has *p_BP _≤ *0.05 (i.e., bicluster 511-512 for which *p_BP _*= 9.85 *E *- 5). In *mirDIP-B*, the total number of biclusters including miR-17 is 26 and, among them, 16 (more than 60%) have a significant *p_BP _*value. This result is even more surprising if we consider the total number of biclusters obtained by the two experiments at all levels of the hierarchy. Indeed, despite the doubling of biclusters containing miR-17, from *mirDIP-A *to *mirDIP-B*, the total number of *mirDIP-B *biclusters is smaller (996) than that of *mirDIP-A *biclusters (1192), with a size decrease in *mirDIP-B *of about 27%. This is due to better precision and recall capabilities provided by the new algorithm, which lead to an improvement of HOCCLUS2's sensitivity in detecting, among all those possible, the miRNA:mRNA biclusters which are more functionally related.

**Table 7:** Biclucters containing members of the miR-17-92 gene cluster family in *mirDIP-A *and *mirDIP-B*.

Level	ID	miRNAs	mRNAs	q	p_BP_	p_MF_
***mirDIP-A *biclusters containing members of miR-17-92**						

L1	412	3	4	0.541	1.0	0.0
	455	8	7	0.608	1.0	1.03E-10
	503	3	57	0.581	1.0	1.0
	511	3	58	0.603	0.40	1.0
	514	5	58	0.619	1.0	0.040

L2	412-514	6	65	0.617	1.0	0.25
	455-503	8	62	0.521	1.0	1.0
	511-512	6	116	0.598	9.85E-5	5.14E-7

L3	208-381-455-503	12	67	0.376	1.0	1.0
	412-514-511-512	6	178	0.603	0.091	5.80E-4

***mirDIP-B *biclusters containing members of miR-17-92**						

L1	181	3	2	0.837	1.0	1.0
	189	3	17	0.865	0.01	1.0
	197	3	2	0.852	1.0	1.0
	294	3	2	0.79	1.0	1.0
	379	3	4	1.0	0.0	0.0
	400	3	6	0.889	0.23E-3	1.0
	405	3	7	0.911	1.0	1.0
	409	3	9	0.891	0.0	0.0
	413	3	9	0.767	0.0	1.0
	415	3	13	0.936	0.0	7.71E-31

L2	181-294	4	4	0.706	1.0	1.0
	189-400	4	19	0.779	0.10E-3	1.0
	197-413	4	11	0.6	0.06	1.0
	379-405	4	11	0.725	0.0	1.0
	409-415	4	22	0.809	0.0	0.0

L3	54-290-197-413	7	23	0.356	1.0	1.0
	160-275-409-415	5	34	0.691	8.90E-31	3.46E-30
	181-294-189-400	5	22	0.673	7.94E-4	1.0
	348-356-379-405	6	17	0.597	4.06E-12	1.0

From an overall evaluation of cohesiveness values, we can see that they are generally higher in *mirDIP-B *biclusters. This result, combined with lower *p_BP _*and *p_MF _*values, indicates a higher biological consistency of biclusters extracted by HOCCLUS2 when exploiting interactions identified by the new algorithm.

Finally, the smaller dimension of biclusters in *mirDIP-B *and the balanced distribution of significant biclusters among the different levels of the HOCCLUS2 hierarchy allow us to interpret better the results. In particular, this provides the necessary information to detect alternative co-targeting of miRNAs on different and potentially co-regulated groups of target genes.

#### Biological evaluation of miR-17-92 biclusters in mirDIP-A and mirDIP-B

In the previous experiments (reported in [7]), we identified a series of highly-ranked biclusters extracted from miRTarBase, containing the members of the miR-17-92 gene cluster family (see Table 8 in [7]). We also extensively discussed miRNA functions and multiple associations that might be consistent with functions and mechanisms of miR-17-92 reported in the literature. We were also able to demonstrate how the functional associations suggested by the analysis of HOCCLUS2 provide new clues on potential cooperative interactions of some members of miR-17-92 with other miRNAs that could be, in turn, the determining factors for a context-specific activity of miR-17-92.

In spite of a good result obtained from miRTarBase, the biological evaluation of miR-17-92 biclusters extracted from mirDIP with the SA-3B setting was quite disappointing. Indeed, as shown in Table 7, mirDIP-A contains only one bicluster including miR-17 with *p_BP _≤ *0.05. This bicluster (i.e., 511-512) groups six different members of the miR-17-92 gene cluster family (i.e., miR-17, miR-93, miR-20a and b, and miR-106a and b) that potentially co-target 116 different genes. Although the analysis of this bicluster with Reactome (see Additional File 1) does not provide a mapping for 68 out of the 116 genes, their over-representation analysis proves to be consistent with many of the known functions of miR-17-92 [52]. However, although significantly better than results obtained in the preliminary analysis of SA, they still return a picture that is too general, because of the high number of target genes included in the bicluster. On the other hand, the unavailability of enough biclusters with a statistical functional significance at different levels of the hierarchy affects the possibility of detecting alternative contributions of each member of the family on specific events or pathways *i) *in different combinations with other members of miR-17-92 in the same bicluster and *ii) *with other members not included in the bicluster.

The analysis of miR-17-92 biclusters in mirDIP-B shows how the new approach helps to overcome these limitations. Indeed, as reported in the previous subsection, the approach presented in this paper has allowed HOCCLUS2 to identify many biclusters with a significant *p_BP _*value. The functional analysis of these biclusters demonstrates that they group together functionally related miRNAs and target genes. A significant example, among many that could be reported, is represented by bicluster 379, that is one of the top-ranked biclusters at level 1 of the hierarchy (see Table 7). This bicluster shows a significant enrichment in the TGF-*β*/BMP pathway, which regulates embryonic and adult cell proliferation and differentiation, and that is a well-known target of miR-17-92. Bicluster 379 groups together TGFBR2, BMPR2, SMAD and PTEN as targets of miR-17, miR-19 and miR-20a, which are members of the miR-17-92 gene cluster. BMPR2 and TGFBR2 are key factors for the activation of TGF-*β*/BMP receptor complexes and for the transduction of the signal from the cell surface to the cytosol. SMAD4 is essential for the transduction of the signal to the nucleus and the transcriptional activation of a series of effectors. PTEN is another key component of the TGF-*β *signaling cascade and, like other genes in this bicluster, it is a validated target of miR-17-92 [53]. This bicluster is particularly interesting because it mimes bicluster 66, obtained in our experiment on miRTarBase data, as reported in [7]. This result is a good indicator of the higher functional cohesiveness that is obtained by the use of the new algorithm on miRNA target site predictions.

Moreover, at level 2 of the hierarchy, bicluster 379 is merged with bicluster 405, which groups together miR-17 and miR-20a (belonging to the miR-17-92 gene cluster), with miR-20b (belonging to the miR-160a-363 gene cluster). As shown in Table 7, the *p_BP _*value of bilcuster 405 is not significant. Indeed, its target genes (i.e., BCL2, CRTC3, MUC17, VEGFA, WDFY2, C6ORF151, KIAA1462) do not show any evident functional relationship. However, they appear functionally related after merging them with genes of bicluster 379. Indeed, analyzing bicluster 379-405 with STRING, we have found that BCL2, CRTC3, VGFA and WDFY2 are included in the interaction network of all the genes in bicluster 379 (see Additional File 2). This result shows a potential cooperation of miR-106a-363 with miR-17-92 in mediating specific events, functionally related to the general control of miR-17-92 on the TGF-*β *signaling pathway, that could be context- or tissue-specific. A further confirmation of this observation comes from the analysis of genes excluded by the interaction network in the STRING analysis, i.e. MUC17, C6ORF151 and KIAA1462. In particular, MUC17 is a membrane mucin that probably plays a role in maintaining homeostasis on mucosal surfaces and that is mainly expressed in the digestive tract. It may conduct signals in response to external stimuli that lead to cellular responses, including proliferation, differentiation, apoptosis or secretion of cellular products, such as other membrane-bound mucin members [54]. According to [54], this gene is a validated target of miR-17, miR-20a, miR-20b. As for C6ORF151 and KIAA1462, the only information that can be retrieved is that C6ORF151 is a nuclear ribonucleoprotein and that KIAA1462 is a junctional protein associated with coronary artery disease. Although these two last genes do not appear to be directly related to the others in the bicluster, we cannot exclude that their potential functional relationships are not detected because of the still poor availability of functional data or of missing annotations in the main web resources. Indeed, as demonstrated for MUC17, neither Reactome nor STRING analysis have been able to detect its functional relationship with other genes in bicluster 319-405. Finally, it is important to underline that MUC17 was not associated with miR-17-92 in the previous analysis [7] of miRTarBase.

Another interesting example that we can provide is represented by bicluster 415. It is another top-ranked bicluster at level 1 of the hierarchy which, similarly to bicluster 379, mimes a bicluster obtained by the previous analysis [7] on miRTarBase, i.e. bicluster 72. Bicluster 415 is highly enriched in genes specifically involved in the cell cycle. Namely, it includes six (i.e., E2F3, RB1, RBL2, CCND2, WEE1, CCND1) out of 13 genes in the mitotic G1-G1/S phases as specific targets of miR-17, miR-20a and miR-106b. In addition, bicluster 415 includes three genes that Reactome does not annotate, that are BECN1, C20ORF82 (i.e., p300 or KAT3B) and FAIM2. Similarly to bicluster 379-405, the over-representation analysis of Reactome significantly maps only the genes involved in the cell cycle (Additional File 3), whereas STRING is able to find functional relationships among 10 out of the 13 genes included in the bicluster (see Additional File 4).

Many other significant examples could be reported, but the discussion of all the biological implications that they highlight would require too much space in the context of the present paper. Just as a last example, other interesting observations arise in the analysis of miR-17-92 biclusters at higher levels of the hierarchy in *mirDIP-B*. Indeed, the functional analysis of biclusters at levels 4, 5 and 6 of the identified hierarchy, that observed a degeneration in *mirDIP-A*, has surprisingly shown in *mirDIP-B *a good distribution and a statistical over-representation in pathways that are perfectly consistent with miR-17-92 known biological functions. What is of more interest is that these biclusters group together miR-17-92 gene cluster members with those belonging to another important miRNA gene cluster, i.e. miR-520. This finding is functionally related to the role of miR-17-92 in the cell cycle, development and differentiation. Indeed, the functional inter-relationship between miR-17-92 and miR-520 has been experimentally demonstrated in a study for investigating the molecular mechanisms responsible for the simultaneous maintenance of human embryonic stem (hES) cells, their self-renewal properties and undifferentiated state [55]. The elucidation of the coordinated activity of miR-17-92 and miR-520 miRNAs, as well as of the regulatory networks that they are able to establish with their target genes, can largely contribute to *i) *the understanding of the physiology of hES cells development and differentiation and to *ii) *the exploitation of their potential as best candidate resources for both cell replacement therapy and development research. The association of miR-17-92 with miR-520 was not detected either in mirDIP-A or in biclusters extracted from miRTarBase.

The conclusions that arise from the reported analysis clearly show the effectiveness of the proposed approach in improving the performance of HOCCLUS2 on mirDIP data under many aspects. In particular, it gives HOCCLUS2 the ability to extract biologically realistic biclusters, which appear more related at different levels of the hierarchy and, more importantly, which represent consistent functional interactions not detected on experimental data. This last aspect demonstrates that, in general, the use of large-scale prediction data of miRNAs target sites can reveal functional connections otherwise impossible to detect from experimental data that are usually context-specific and, hence, lack a comprehensive view of the system.

## Conclusions

In this work we have investigated the possibility to improve the reliability of miRNA:miRNA predicted interactions. In particular, we have proposed the application of a machine learning technique, in order to learn to combine the outputs of several prediction algorithms. Since the domain in hand is characterized by the availability of a small number of labeled examples and a very large number of unlabeled examples, the proposed approach relies on a semi-supervised algorithm, which exploits information conveyed by both positive/labeled and unlabeled examples. Moreover, the unbalancing between the number of labeled and unlabeled examples is tackled by adopting an ensemble learning approach.

The effectiveness of the proposed approach has been evaluated according to many criteria. First, the predictive performance of the proposed approach on an independent set of experimentally validated interactions is higher than that obtained by single prediction algorithms and by other baseline combination strategies. Second, HOCCLUS2 has been applied to different datasets of predicted interactions, according to different combination strategies. Results prove that the proposed approach is able to better filter out false positives and allows HOCCLUS2 to focus on only reliable interactions. This leads to the identification of more precise and significant interaction networks. Finally, an in depth biological analysis of some examples of extracted biclusters has been performed. This analysis shows how the proposed approach leads to the discovery of a hierarchy with a balanced distribution of significant biclusters among different levels, which, in general, improves the possibility to interpret results from a biological viewpoint. Moreover, we focused on biclusters that group together members of the miR-17-92 gene cluster family. In this case, we have observed that the functional analysis of biclusters at higher levels of the hierarchy, that appears highly degenerated with the other combination strategies, surprisingly shows a good distribution and a statistical over-representation in pathways that are perfectly consistent with the known miR-17-92 biological functions. Above all, HOCCLUS2 was able to group together, at high levels of the hierarchy, members of the miR-17-92 gene cluster with those belonging to the miR-520 gene cluster. Its relation with mir-17-92 has been experimentally proved and was not identified either from experimentally validated interactions or from predicted interactions originating from other combination strategies.

These results prove that the contribution of the proposed approach is, in general, fundamental in the computational discovery of reliable miRNA:mRNA interactions. In particular, it is essential for the extraction of biological realistic networks of interactions between miRNAs and their target genes from prediction data. This last aspect opens up the possibility to expand the application of HOCCLUS2 on a "genome-scale" dimension for a comprehensive reconstruction of all the possible multiple interactions established by miRNAs to regulate the expression of gene networks, which are otherwise impossible to identify when only experimentally validated interactions are considered.

For future work, we intend to investigate the possibility of integrating low-level features in the learning phase, with the aim of improving the predictive capabilities of the proposed approach.

## Availability of supporting data

**Project Home Page: **http://www.di.uniba.it/~ceci/micFiles/systems/semisupervised_HOCCLUS2/index.html

**Available resources: **The proposed system, all the datasets and all the obtained results.

## List of abbreviations

5' UTR: 5' Untranslated Region; AUC: Area Under the ROC Curve; BP: Biological Process; CDS: Coding Sequence; CLIP-Seq: Cross-Linking Immunoprecipitation-High-Throughput Sequencing; FPr: False Positive rate; hES cells: Human Embryonic Stem Cells; MF: Molecular Function; miRNA: microRNA; mRNA: messenger RNA; PARE: Parallel Analysis of RNA ends; RISC: RNAi-Induced Silencing Complex; SA: Score Averaging; SA-3B: Score averaging - Three Best; SVM: Support Vector Machine; TPr: True Positive rate; WSA-3B: Weighted score averaging - Three Best.

## Competing interests

The authors declare that they have no competing interests.

## Authors' contributions

MC and GP contributed to the definition of the method. DD contributed to the conception of the biological investigation. GP and MC contributed to the software design. GP and DD took care of the review and selection of bioinformatic resources. GP implemented the system and ran the experiments. DD performed the biological analysis and validation of the results. GP and MC performed the analysis of the results, from the computer science point of view. MC, GP and DD contributed to the manuscript drafting. MC, GP, DD and DM contributed to the manuscript finalization. DM and MC supervised the study. All the authors read and approved the final manuscript.

## Supplementary Material

Additional file 1 **Reactome mapping of bicluster 511-512 in mirDIP-A**. Click here for file

Additional file 2 **STRING network of bicluster 379-405 in mirDIP-B**. Click here for file

Additional file 3 **Reactome mapping of bicluster 415 in mirDIP-B**. Click here for file

Additional file 4 **STRING network of bicluster 415 in mirDIP-B**. Click here for file

## Declarations

Publication of this article was supported by the project "MBLab: The Molecular Biodiversity LABoratory Initiative" (MIUR DM 19410).

This article has been published as part of *BMC Bioinformatics *Volume 15 Supplement 1, 2014: Integrated Bio-Search: Selected Works from the 12th International Workshop on Network Tools and Applications in Biology (NETTAB 2012). The full contents of the supplement are available online at http://www.biomedcentral.com/bmcbioinformatics/supplements/15/S1.
