# Supplementary figures and images for: Integrating microRNA target predictions for the discovery of gene regulatory networks: a semi-supervised ensemble learning approach
Source: BMC Bioinformatics. 2014 Jan 10;15(Suppl 1):S4. doi: 10.1186/1471-2105-15-S1-S4 (PMC4015287; doi:10.1186/1471-2105-15-S1-S4)

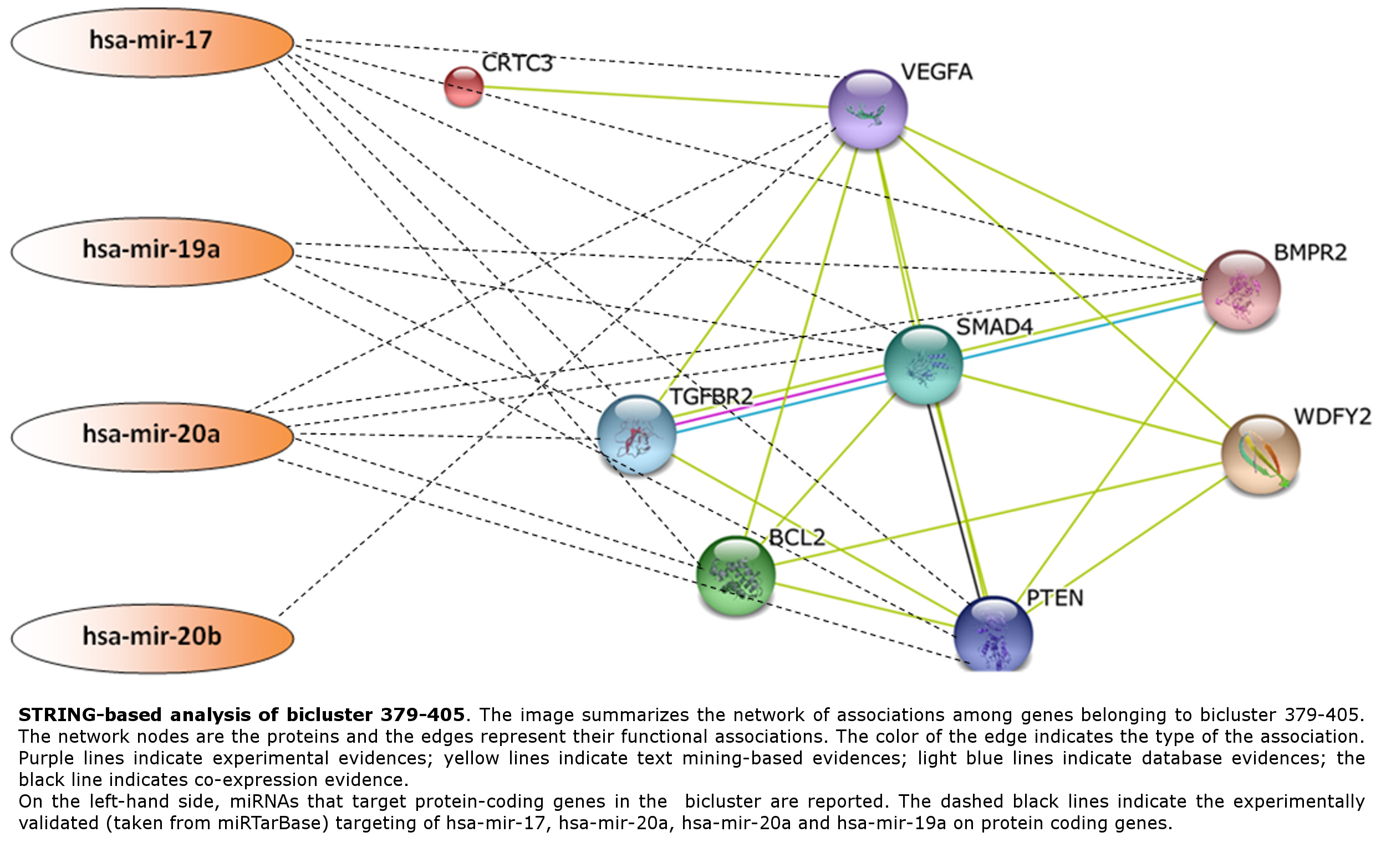

Supplement: Additional file 2 — STRING network of bicluster 379-405 in mirDIP-B. [file 1471-2105-15-S1-S4-S2.png]

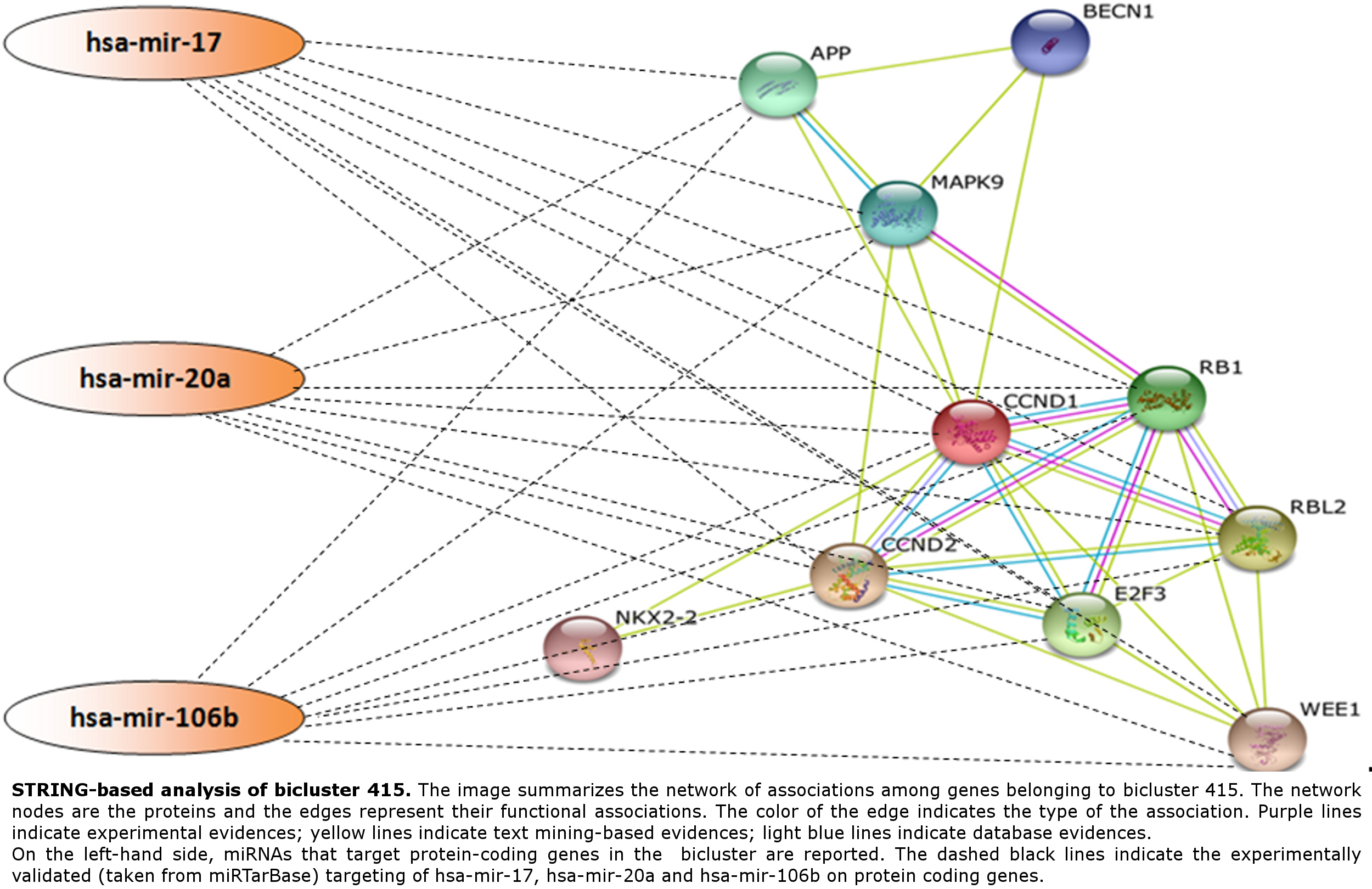

Supplement: Additional file 4 — STRING network of bicluster 415 in mirDIP-B. [file 1471-2105-15-S1-S4-S4.png]
